# Supplementary material for: Analysis of PPARγ Signaling Activity in Psoriasis
Source: Int J Mol Sci. 2021 Aug 10;22(16):8603. doi: 10.3390/ijms22168603 (PMC8395241; doi:10.3390/ijms22168603)
Supplement: Supplementary file 1 [file ijms-22-08603-s001.zip › Supplemental materials_Analysis of PPARg signaling activity in psoriasis/Pathway models/Models images and html files/Model of PPARG related pathways in psoriasis (short version)/388.html]

AGER


# Protein AGER

|  |  |
| --- | --- |
| URN | urn:agi-llid:177 |
| Total Entities | 0 |
| Connectivity | 4332 |
| Name | AGER |
| Description | advanced glycosylation end product-specific receptor |
| Notes | The advanced glycosylation end product (AGE) receptor encoded by this gene is a member of the immunoglobulin superfamily of cell surface receptors. It is a multiligand receptor, and besides AGE, interacts with other molecules implicated in homeostasis, development, and inflammation, and certain diseases, such as diabetes and Alzheimer's disease. Many alternatively spliced transcript variants encoding different isoforms, as well as non-protein-coding variants, have been described for this gene (PMID:18089847). [provided by RefSeq, May 2011] |
| Primary Cell Localization | Plasma membrane |
| Class | Receptor |
| ObjectType | Protein |

---

|  |  |
| --- | --- |
| Pathway | Osteoarthritis |
|  | Extracellular Matrix Reformation in Aging |
|  | Toll-like Receptors in Sterile Inflammation |
|  | Toll-like Receptor Independent Sterile Inflammation |
|  | Proteins Involved in Atherosclerosis |
|  | RAGE/AGER and S100 Proteins in Cardiovascular Injury |
|  | Proteins Involved in Arterial Hypertension |
|  | Myocardial Infarction |
|  | Proteins Involved in Myocardial Ischemia |
|  | Dendritic Cells Function in Psoriasis |
|  | Dendritic Cell Activation in Systemic Lupus Erythematosis |
|  | Proteins Involved in Diabetes Mellitus Type 2 |
|  | Advanced Glycation End Products Pathway in Diabetic Microangiopathy |
|  | Peripheral Tissue Microangiopathy |
|  | Tubular Cell Dysfunction in Progressive Diabetic Nephropathy |
|  | Podocyte Dysfunction in Diabetic Nephropathy |
|  | Endothelial Cell Dysfunction in Early Diabetic Nephropathy |
|  | Proteins Involved in Diabetic Nephropathy |
|  | Mesangial Cell Dysfunction in Diabetic Nephropathy |
|  | Proteins Involved in Diabetic Neuropathy |
|  | Oxidative Stress in Diabetic Neuropathy |
|  | Microangiopathy in Diabetic Neuropathy |
|  | Proliferative Diabetic Retinopathy |
|  | AGE Related Insulin Resistance in Skeletal Muscles |
|  | Proteins Involved in non-Alcoholic Fatty Liver Disease |
|  | Proteins Involved in Osteoarthritis |
|  | Dendritic Cell Dysfunction in Psoriatic Arthritis |
|  | Diabetes Induced Periodontitis |
|  | Proteins Involved in Periodontitis |
|  | Mevalonate Kinase Deficiency |
|  | Proteins Involved in Alzheimer's Disease |
|  | Amyloid beta Traffic and Degradation in Extracellular Matrix in Alzheimer's Disease |
|  | Proteins Involved in Amyotrophic Lateral Sclerosis |
|  | Proteins Involved in Endometriosis |
|  | HMGB1/RAGE Signaling in Middle Ear Cholesteatoma |
|  | Cholesteatoma |
|  | Proteins Involved in Age-Related Macular Degeneration |
|  | Proteins Involved in Cataract |
|  | Immune System Role in Otitis Media |
|  | Alveolar Macrophages Dysfunction in COPD |
|  | Proteins Involved in Chronic Obstructive Pulmonary Disease |
|  | T-Cell Cytotoxic Activation |
|  | Alveolar Epithelial Cell Dysfunction in COPD |
|  | Advanced Glycation End Products in Diabetes or Age Related Erectile Dysfunction |
|  | S100P Expression Target |
|  | HMGB1 Expression Targets |
|  | TTR Expression Targets |
|  | S100A Expression Targets |
|  | S100B Expression Targets |
|  | Elevated Receptors -> Expression Targets in Adipose Tissue |
|  | Elevated Receptors -> Expression Targets in Bone and Joint |
|  | Elevated Receptors -> Expression Targets in Blood vessel |
|  | Elevated Receptors -> Expression Targets in Blood Vessel |
|  | IDO1 in Cancer Immune Escape |
|  | AGER -> NF-kB Signaling |
|  | AGER -> CREB/SP1 Signaling |
|  | Fibronectin Expression Targets |
|  | Immunoglobulin Superfamily -> Expression Targets in Lymphoid System and Blood |
|  | Elevated Receptors -> Expression Targets in Nerve Tissue |
|  | AGT -> CREB Expression Targets |
|  | Elevated Receptors -> Expression Targets in Lung |
|  | TLR4 -> AP-1/EGR1/HIF1A Expression Targets |
|  | IL1B Expression Targets |
|  | prarg negative regulators, ps-positive |
|  | prarg neg,ukn expres targets, ps-positive |
|  | PPARG negative regulators |
|  | PPARG negative targets |
|  | pparg expr target\_regulators |
|  | Neighbors of disease exacerbation |
|  | Neighbors of tumor progression |
|  | Neighbors of cancer progression |
|  | Neighbors of inflammatory status |
|  | Neighbors of overall survival |
|  | Neighbors of disease severity |
|  | Neighbors of tumor size |
|  | Neighbors of lesion size |
|  | Neighbors of mortality |
|  | Neighbors of survival rate |
|  | Neighbors of infarct size |
|  | Neighbors of survival time |
|  | Neighbors of functional recovery |
|  | Neighbors of insulin sensitivity |
|  | Neighbors of bacterial load |
|  | Neighbors of cancer survival |
|  | Neighbors of therapeutic efficacy |
|  | Neighbors of lethality |
|  | Neighbors of disease-free survival |
|  | Neighbors of treatment outcome |
|  | Neighbors of bone mass |
|  | Neighbors of body weight |
|  | Neighbors of body weight gain |
|  | Neighbors of cardiovascular risk |
|  | Neighbors of microvessel density |
|  | Neighbors of tumor promotion |
|  | Neighbors of glucose tolerance |
|  | Neighbors of morbidity |
|  | Neighbors of fibrosis progression |
|  | Neighbors of mortality rate |
|  | Neighbors of exhaustion |
|  | Neighbors of bone mineral density |
|  | Neighbors of lesion area |
|  | Neighbors of cell density |
|  | Neighbors of apoptosis rate |
|  | Neighbors of shear stress |
|  | Neighbors of viral load |
|  | Neighbors of blood pressure |
|  | Neighbors of leukocyte count |
|  | Neighbors of vascular reactivity |
|  | Neighbors of vascular leakage |
|  | Neighbors of body mass index |
|  | Neighbors of cancer risk |
|  | Neighbors of systolic blood pressure |
|  | Neighbors of blood glucose |
|  | Neighbors of ejection fraction |
|  | Neighbors of median survival |
|  | Neighbors of renal hypertrophy |
|  | Neighbors of fasting plasma glucose |
|  | Neighbors of osteoclast number |
|  | Neighbors of energy expenditure |
|  | Neighbors of glucose level |
|  | Neighbors of vascular tone |
|  | Neighbors of breast cancer risk |
|  | Neighbors of cell permeability |
|  | Neighbors of infarct area |
|  | Neighbors of pathological response |
|  | Neighbors of metabolic response |
|  | Neighbors of insulin level |
|  | Neighbors of comorbidity |
|  | Neighbors of carotid intima-media thickness |
|  | Neighbors of fractional shortening |
|  | Neighbors of disease exacerbation |
|  | Neighbors of tumor progression |
|  | Neighbors of cancer progression |
|  | Neighbors of inflammatory status |
|  | Neighbors of overall survival |
|  | Neighbors of disease severity |
|  | Neighbors of tumor size |
|  | Neighbors of lesion size |
|  | Neighbors of mortality |
|  | Neighbors of survival rate |
|  | Neighbors of infarct size |
|  | Neighbors of survival time |
|  | Neighbors of functional recovery |
|  | Neighbors of insulin sensitivity |
|  | Neighbors of bacterial load |
|  | Neighbors of cancer survival |
|  | Neighbors of therapeutic efficacy |
|  | Neighbors of lethality |
|  | Neighbors of disease-free survival |
|  | Neighbors of treatment outcome |
|  | Neighbors of bone mass |
|  | Neighbors of body weight |
|  | Neighbors of body weight gain |
|  | Neighbors of cardiovascular risk |
|  | Neighbors of microvessel density |
|  | Neighbors of tumor promotion |
|  | Neighbors of glucose tolerance |
|  | Neighbors of morbidity |
|  | Neighbors of fibrosis progression |
|  | Neighbors of mortality rate |
|  | Neighbors of exhaustion |
|  | Neighbors of bone mineral density |
|  | Neighbors of lesion area |
|  | Neighbors of cell density |
|  | Neighbors of apoptosis rate |
|  | Neighbors of shear stress |
|  | Neighbors of viral load |
|  | Neighbors of blood pressure |
|  | Neighbors of leukocyte count |
|  | Neighbors of vascular reactivity |
|  | Neighbors of vascular leakage |
|  | Neighbors of body mass index |
|  | Neighbors of cancer risk |
|  | Neighbors of systolic blood pressure |
|  | Neighbors of blood glucose |
|  | Neighbors of ejection fraction |
|  | Neighbors of median survival |
|  | Neighbors of renal hypertrophy |
|  | Neighbors of fasting plasma glucose |
|  | Neighbors of osteoclast number |
|  | Neighbors of energy expenditure |
|  | Neighbors of glucose level |
|  | Neighbors of vascular tone |
|  | Neighbors of breast cancer risk |
|  | Neighbors of cell permeability |
|  | Neighbors of infarct area |
|  | Neighbors of pathological response |
|  | Neighbors of metabolic response |
|  | Neighbors of insulin level |
|  | Neighbors of comorbidity |
|  | Neighbors of carotid intima-media thickness |
|  | Neighbors of fractional shortening |
|  | Neighbors of cell development |
|  | Neighbors of immune response |
|  | Neighbors of cell population |
|  | Neighbors of T-cell development |
|  | Neighbors of inflammatory response |
|  | Neighbors of wound healing |
|  | Neighbors of angiogenesis |
|  | Neighbors of innate immune response |
|  | Neighbors of cell function |
|  | Neighbors of macrophage activation |
|  | Neighbors of cell formation |
|  | Neighbors of adaptive immune response |
|  | Neighbors of osteoclast development |
|  | Neighbors of epithelial to mesenchymal transition |
|  | Neighbors of immunity |
|  | Neighbors of cell proliferative response |
|  | Neighbors of chemotaxis |
|  | Neighbors of dendritic cell differentiation |
|  | Neighbors of cell survival |
|  | Neighbors of osteoclast differentiation |
|  | Neighbors of cell phenotype |
|  | Neighbors of T-cell proliferation |
|  | Neighbors of tissue remodeling |
|  | Neighbors of cell adhesion |
|  | Neighbors of T-cell activation |
|  | Neighbors of endothelial cell proliferation |
|  | Neighbors of fibrogenesis |
|  | Neighbors of tumor growth |
|  | Neighbors of dendritic cell development |
|  | Neighbors of tissue repair |
|  | Neighbors of cell count |
|  | Neighbors of cell infiltration |
|  | Neighbors of cell interaction |
|  | Neighbors of SMC proliferation |
|  | Neighbors of lumen formation |
|  | Neighbors of endothelial cell migration |
|  | Neighbors of adipogenesis |
|  | Neighbors of lipid storage |
|  | Neighbors of cell damage |
|  | Neighbors of pregnancy |
|  | Neighbors of monocyte differentiation |
|  | Neighbors of aging |
|  | Neighbors of cell motility |
|  | Neighbors of bone resorption |
|  | Neighbors of monocyte migration |
|  | Neighbors of fibroblast proliferation |
|  | Neighbors of adipocyte differentiation |
|  | Neighbors of macrophage migration |
|  | Neighbors of microglial activation |
|  | Neighbors of neutrophil recruitment |
|  | Neighbors of regeneration |
|  | Neighbors of cell migration |
|  | Neighbors of cancer growth |
|  | Neighbors of stem cell proliferation |
|  | Neighbors of smooth muscle cell migration |
|  | Neighbors of hepatic regeneration |
|  | Neighbors of transendothelial migration |
|  | Neighbors of phagocytosis |
|  | Neighbors of immune system activation |
|  | Neighbors of leukocyte migration |
|  | Neighbors of leukocyte recruitment |
|  | Neighbors of osteoclast formation |
|  | Neighbors of monocyte recruitment |
|  | Neighbors of cell invasion |
|  | Neighbors of heart function |
|  | Neighbors of bone remodeling |
|  | Neighbors of ossification |
|  | Neighbors of immune system function |
|  | Neighbors of lung development |
|  | Neighbors of autophagy |
|  | Neighbors of monocyte adhesion |
|  | Neighbors of transcription activation |
|  | Neighbors of senescence |
|  | Neighbors of tumor immunity |
|  | Neighbors of hepatocyte proliferation |
|  | Neighbors of cell transdifferentiation |
|  | Neighbors of neuroprotection |
|  | Neighbors of stem cell differentiation |
|  | Neighbors of endothelial cell function |
|  | Neighbors of neuronal death |
|  | Neighbors of cell growth |
|  | Neighbors of ROS generation |
|  | Neighbors of cancer cell growth |
|  | Neighbors of sensitization |
|  | Neighbors of hemato-encephalic barrier |
|  | Neighbors of cellular immune response |
|  | Neighbors of cellular senescence |
|  | Neighbors of colony formation |
|  | Neighbors of macrophage differentiation |
|  | Neighbors of cell differentiation |
|  | Neighbors of stem cell migration |
|  | Neighbors of cell homeostasis |
|  | Neighbors of psoriasis |
|  | Neighbors of inflammatory disease |
|  | Neighbors of rheumatoid arthritis |
|  | Neighbors of atherosclerosis |
|  | Neighbors of fibrosis |
|  | Neighbors of colitis |
|  | Neighbors of arthritis |
|  | Neighbors of inflammatory bowel disease |
|  | Neighbors of injury |
|  | Neighbors of atherogenesis |
|  | Neighbors of vascular remodeling |
|  | Neighbors of metastasis |
|  | Neighbors of carcinogenesis |
|  | Neighbors of multiple sclerosis |
|  | Neighbors of chronic inflammation |
|  | Neighbors of inflammation |
|  | Neighbors of tumor microenvironment |
|  | Neighbors of pathological angiogenesis |
|  | Neighbors of obesity |
|  | Neighbors of vascular disease |
|  | Neighbors of diabetes mellitus |
|  | Neighbors of asthma |
|  | Neighbors of pneumonia |
|  | Neighbors of hyperplasia |
|  | Neighbors of osteoarthritis |
|  | Neighbors of liver fibrosis |
|  | Neighbors of chronic obstructive pulmonary disease |
|  | Neighbors of myocardial infarction |
|  | Neighbors of leukocyte infiltration |
|  | Neighbors of reperfusion injury |
|  | Neighbors of airway inflammation |
|  | Neighbors of hepatitis |
|  | Neighbors of insulin resistance |
|  | Neighbors of infection |
|  | Neighbors of vascular injury |
|  | Neighbors of renal injury |
|  | Neighbors of preeclampsia |
|  | Neighbors of macrophage infiltration |
|  | Neighbors of liver injury |
|  | Neighbors of neuroinflammation |
|  | Neighbors of sepsis |
|  | Neighbors of death |
|  | Neighbors of type 1 diabetes |
|  | Neighbors of encephalomyelitis |
|  | Neighbors of autoimmune disease |
|  | Neighbors of ischemic stroke |
|  | Neighbors of dermatitis |
|  | Neighbors of neutrophil infiltration |
|  | Neighbors of heart disease |
|  | Neighbors of diabetic nephropathy |
|  | Neighbors of lung injury |
|  | Neighbors of immunopathology |
|  | Neighbors of hepatocellular carcinoma |
|  | Neighbors of autoimmunity |
|  | Neighbors of acute lung injury |
|  | Neighbors of gastric cancer |
|  | Neighbors of atherosclerotic plaque |
|  | Neighbors of experimental autoimmune encephalomyelitis |
|  | Neighbors of colorectal cancer |
|  | Neighbors of cardiac remodeling |
|  | Neighbors of breast cancer |
|  | Neighbors of ischemia |
|  | Neighbors of systemic lupus erythematosus |
|  | Neighbors of melanoma |
|  | Neighbors of collagen-induced arthritis |
|  | Neighbors of cartilage degeneration |
|  | Neighbors of interstitial fibrosis |
|  | Neighbors of neoplasm invasion |
|  | Neighbors of kidney disease |
|  | Neighbors of heart failure |
|  | Neighbors of lung disease |
|  | Neighbors of pulmonary fibrosis |
|  | Neighbors of cardiovascular disease |
|  | Neighbors of stroke |
|  | Neighbors of cancer |
|  | Neighbors of cerebral ischemia |
|  | Neighbors of type 2 diabetes |
|  | Neighbors of neoplasm |
|  | Neighbors of endometriosis |
|  | Neighbors of neointima |
|  | Neighbors of vasculitis |
|  | Neighbors of atrophy |
|  | Neighbors of diet-induced obesity |
|  | Neighbors of respiratory hypersensitivity |
|  | Neighbors of myocardial fibrosis |
|  | Neighbors of synovitis |
|  | Neighbors of coronary artery disease |
|  | Neighbors of rupture |
|  | Neighbors of endothelial cell dysfunction |
|  | Neighbors of liver disease |
|  | Neighbors of prostate cancer |
|  | Neighbors of hypertrophy |
|  | Neighbors of pancreatic cancer |
|  | Neighbors of hypertension |
|  | Neighbors of intimal hyperplasia |
|  | Neighbors of allograft rejection |
|  | Neighbors of proteinuria |
|  | Neighbors of vascular smooth muscle cell |
|  | Neighbors of umbilical vein endothelial cell |
|  | Neighbors of monocyte |
|  | Neighbors of synoviocyte |
|  | Neighbors of macrophage |
|  | Neighbors of adipocyte |
|  | Neighbors of fibroblast |
|  | Neighbors of osteoblast |
|  | Neighbors of pneumocyte |
|  | Neighbors of microglia |
|  | Neighbors of kidney mesangial cell |
|  | Neighbors of endothelial cell |
|  | Neighbors of inflammatory cell |
|  | Neighbors of neutrophil |
|  | Neighbors of mesothelial cell |
|  | Neighbors of capillary endothelial cell |
|  | Neighbors of immunocompetent cell |
|  | Neighbors of cardiac myocyte |
|  | Neighbors of stem cell |
|  | Neighbors of leukocyte |
|  | Neighbors of chondrocyte |
|  | Neighbors of spleen cell |
|  | Neighbors of neuroglia |
|  | Neighbors of podocyte |
|  | Neighbors of pericyte |
|  | Neighbors of phagocyte |
|  | Neighbors of lung |
|  | Neighbors of liver |
|  | Neighbors of kidney |
|  | Neighbors of skin |
|  | Neighbors of intestine |
|  | Neighbors of lymph node |
|  | Neighbors of airway |
|  | Neighbors of blood vessel |
|  | Neighbors of heart |
|  | Neighbors of aorta |
|  | Neighbors of microvessel |
|  | Neighbors of bone |
|  | Neighbors of placenta |
|  | Neighbors of carotid artery |
|  | Neighbors of esophagus |
|  | Neighbors of periodontium |
|  | Neighbors of brain |
|  | Neighbors of ovary |
|  | Neighbors of stomach |
|  | Neighbors of islets of Langerhans |
|  | Neighbors of pancreas |
|  | Neighbors of central nervous system |
|  | Neighbors of kidney glomerulus |
|  | Neighbors of colorectal region |
|  | Neighbors of alveolus |
|  | Neighbors of coronary artery |
|  | Neighbors of omentum |
|  | Neighbors of intervertebral disc |
|  | Neighbors of nervous system |
|  | Neighbors of nerve |
|  | Neighbors of NF-kB family |
|  | Neighbors of oxidized LDL |
|  | Neighbors of PPAR |
|  | Neighbors of NFKBI |
|  | Neighbors of mitogen-activated protein kinase |
|  | Neighbors of TLR |
|  | Neighbors of PKA |
|  | Neighbors of LDL |
|  | Neighbors of AMPK |
|  | Neighbors of estrogen receptor |
|  | Neighbors of T-cell receptor |
|  | Neighbors of HIF-1 |
|  | Neighbors of aldehyde reductase |
|  | Neighbors of Ang II receptor |
|  | Neighbors of NOS |
|  | Neighbors of class nuclear receptor with C4 zinc fingers |
|  | Neighbors of non-coding RNA |
|  | Neighbors of gonadotropins |
|  | Neighbors of AHR subfamily |
|  | Neighbors of angiotensin receptor |
|  | Model of PPARG signaling in psoriasis |
|  | PPARG negative regulators and targets |
|  | Model of PPARG related pathways in psoriasis (short version) |
|  | AGER feed forward loop in innate immunity |
|  | Dendritic Cell Activation in COVID19 |
|  | Overview. Diseases of circulatory system (chapter 8) |
|  | Overview. Diseases of the nervous system (chapter 5) |
|  | Overview. Diseases of the respiratory (chapter 9) |
|  | 3\_Arterial wall inflammation and calcification in atherosclerosis |
|  | 1\_Cigarette smoke causes dysfunction and production of inflammatory mediators in alveolar epithelial cells |
|  | 3\_Role of alveolar macrophages in pulmonary tissue destruction in COPD |
|  | 4\_1\_Polymorphisms associated with inflammatory bowel diseases |
|  | 1\_Microangiopathy in diabetic neuropathy |
|  | 2\_Neuron and Schwann cell death |
|  | 1\_cd\_Amyloid-beta in Alzheimer's disease |
|  | drug-target moltransport |
|  | Model of PPARG signaling in psoriais (tested) |
|  | mapk1 direct regulators |

---

|  |  |
| --- | --- |
| Group | Genes with Mutations in GWA Studies of Asthma |

---

|  |  |
| --- | --- |
| MedScan ID | 177 |

---

|  |  |
| --- | --- |
| LocusLink ID | 177 |
|  | 11596 |
|  | 81722 |

---

|  |  |
| --- | --- |
| Alias | receptor for advanced glycosylation end products |
|  | sRAGE5 |
|  | advanced glycation end-product receptor |
|  | receptor for advanced glycosylation endproducts |
|  | advanced glycation end-products receptor |
|  | RAGE isoform delta |
|  | advanced glycosylation end product-specific receptor variant 4 |
|  | AGE receptor |
|  | advanced glycosylation end product-specific receptors |
|  | advanced glycosylation endproduct receptor |
|  | soluble RAGE variant 2 |
|  | RAGE isoform sRAGE-delta |
|  | sRAGE |
|  | OTTHUMP00000029155 |
|  | advanced glycation endproduct receptor |
|  | soluble RAGE variant 1 |
|  | advanced glycation end product-specific receptor |
|  | sRAGE1 |
|  | receptor for advanced glycosylation end product |
|  | MGC22357 |
|  | advanced glycosylation end product-specific receptor |
|  | receptor for advanced glycation end products |
|  | advanced glycosylation end product-specific receptor variant sRAGE2 |
|  | receptor for advanced glycation endproducts |
|  | soluble RAGE variant 3 |
|  | advanced glycosylation end product-specific receptor variant 3 |
|  | OTTHUMP00000174885 |
|  | RAGE isoform NtRAGE-delta |
|  | receptor for advanced glycosylation endproduct |
|  | advanced glycosylation end product-specific receptor variant 1 |
|  | advanced glycosylation end product-specific receptor variant sRAGE1 |
|  | SCARJ1 |
|  | DAMA-358M23.4 |
|  | advanced glycosylation end product-specific receptor variant 2 |
|  | RAGE |
|  | advanced glycation end product-specific receptors |
|  | advanced glycosylation end product receptor |
|  | receptor for advanced glycation end-products variant 20 |
|  | soluble RAGE |
|  | OTTHUMP00000029156 |
|  | advanced glycation endproducts receptor |
|  | receptor for advanced glycation endproduct |
|  | soluble RAGE variant 5 |
|  | AGER |
|  | soluble RAGE variant 4 |
|  | sRAGE3 |
|  | sRAGE4 |
|  | advanced glycosylation end product-specific receptor variant 5 |
|  | sRAGE2 |

---

|  |  |
| --- | --- |
| GO ID | 0044548 |
|  | 0050785 |
|  | 0001540 |
|  | 0044877 |
|  | 0005044 |
|  | 0038023 |
|  | 0004888 |
|  | 0048143 |
|  | 0007166 |
|  | 1904646 |
|  | 0006897 |
|  | 0010255 |
|  | 0050930 |
|  | 0006954 |
|  | 0045087 |
|  | 0007611 |
|  | 0001774 |
|  | 0090647 |
|  | 1903523 |
|  | 1904597 |
|  | 0032693 |
|  | 1900453 |
|  | 1900272 |
|  | 0031175 |
|  | 0070374 |
|  | 0046330 |
|  | 0043507 |
|  | 0051092 |
|  | 1901224 |
|  | 0042104 |
|  | 1902961 |
|  | 0045080 |
|  | 2001200 |
|  | 1904472 |
|  | 0034116 |
|  | 0050725 |
|  | 0032735 |
|  | 0045410 |
|  | 0071639 |
|  | 2000439 |
|  | 1900745 |
|  | 0001934 |
|  | 0042535 |
|  | 0072657 |
|  | 2000514 |
|  | 0051101 |
|  | 1901222 |
|  | 0001914 |
|  | 1900271 |
|  | 1900744 |
|  | 0150003 |
|  | 0048167 |
|  | 1904645 |
|  | 0001666 |
|  | 0009611 |
|  | 0045056 |
|  | 0016324 |
|  | 0030054 |
|  | 0009986 |
|  | 0005576 |
|  | 0001650 |
|  | 0005887 |
|  | 0005886 |
|  | 0098794 |
|  | 1904599 |
|  | 0008201 |
|  | 0070379 |
|  | 0007259 |
|  | 0014002 |
|  | 0055074 |
|  | 1904604 |
|  | 0007162 |
|  | 0030336 |
|  | 0032966 |
|  | 0010596 |
|  | 0001937 |
|  | 0001933 |
|  | 0023057 |
|  | 1904605 |
|  | 0043065 |
|  | 0061890 |
|  | 0010508 |
|  | 0030335 |
|  | 2000353 |
|  | 0010718 |
|  | 0010763 |
|  | 0048146 |
|  | 0010628 |
|  | 0050729 |
|  | 1903980 |
|  | 0043525 |
|  | 1901216 |
|  | 0060100 |
|  | 1901018 |
|  | 2000379 |
|  | 0023056 |
|  | 0014911 |
|  | 0048661 |
|  | 2000676 |
|  | 0050727 |
|  | 0030424 |
|  | 0009925 |
|  | 0005737 |
|  | 0005829 |
|  | 0009897 |
|  | 0005615 |
|  | 0016021 |
|  | 0016020 |
|  | 0043025 |
|  | 0005634 |
|  | 0007420 |
|  | 0035690 |
|  | 0071398 |
|  | 0071333 |
|  | 0070301 |
|  | 0071407 |
|  | 0009100 |
|  | 0030324 |
|  | 0033689 |
|  | 0014823 |
|  | 0009750 |
|  | 0033595 |
|  | 0055093 |
|  | 0051595 |
|  | 0072714 |
|  | 0033189 |
|  | 0060290 |
|  | 0042493 |
|  | 0005515 |
|  | 0006898 |
|  | 0004872 |
|  | 0046483 |
|  | 0033687 |

---

|  |  |
| --- | --- |
| KEGG ID | hsa:177 |
|  | mmu:11596 |
|  | rno:81722 |

---

|  |  |
| --- | --- |
| Organism | Homo sapiens {Organism urn:agi-taxid:9606} |
|  | Mus musculus {Organism urn:agi-taxid:10090} |
|  | Rattus norvegicus {Organism urn:agi-taxid:10116} |
|  | Homo sapiens |
|  | Mus musculus |
|  | Rattus norvegicus |

---

|  |  |
| --- | --- |
| Mouse chromosome position | 17 |

---

|  |  |
| --- | --- |
| OMIM ID | 600214 |

---

|  |  |
| --- | --- |
| Rat chromosome position | 20p12 |

---

|  |  |
| --- | --- |
| Hugo ID | 320 |
|  | HGNC:320 |

---

|  |  |
| --- | --- |
| Human chromosome position | 6p21.32 |
|  | 6p21.3 |

---

|  |  |
| --- | --- |
| Swiss-Prot Accession | A0A1U9X785 |
|  | B4DNX3 |
|  | Q15109 |
|  | Q15109.1 |
|  | Q62151 |
|  | Q62151.2 |
|  | Q6MG86 |
|  | A0A0G2K218 |
|  | Q63495.1 |
|  | A2BFI7 |
|  | A6NKF0 |
|  | A7Y2U9 |
|  | B0V176 |
|  | Q15279 |
|  | Q3L1R4 |
|  | Q3L1R5 |
|  | Q3L1R6 |
|  | Q3L1R7 |
|  | Q3L1R8 |
|  | Q3L1S0 |
|  | Q86SN1 |
|  | Q9H2X7 |
|  | Q9Y3R3 |
|  | V5R6A3 |
|  | C5H3H4 |
|  | C5H3H5 |
|  | C5H3H7 |
|  | C5H3I0 |
|  | C5H7W3 |
|  | C5H7W4 |
|  | C5H7W5 |
|  | C5H7W6 |
|  | C5H7W7 |
|  | C5H7W8 |
|  | C5H7W9 |
|  | O35444 |
|  | Q2PGG1 |
|  | V5R4Y0 |
|  | Q63495 |

---

|  |  |
| --- | --- |
| PIR ID | I61596 |
|  | T09062 |

---

|  |  |
| --- | --- |
| GenBank ID | NC\_000006 |
|  | NR\_038190 |
|  | XR\_001743190 |
|  | XR\_001743189 |
|  | NM\_001206929 |
|  | NP\_001193858 |
|  | NM\_001206934 |
|  | NP\_001193863 |
|  | NM\_001206932 |
|  | NP\_001193861 |
|  | NM\_172197 |
|  | NP\_751947 |
|  | NM\_001206954 |
|  | NP\_001193883 |
|  | NM\_001206936 |
|  | NP\_001193865 |
|  | NM\_001136 |
|  | NP\_001127 |
|  | NM\_001206966 |
|  | NP\_001193895 |
|  | NM\_001206940 |
|  | NP\_001193869 |
|  | XM\_017010328 |
|  | XP\_016865817 |
|  | NT\_167244 |
|  | NT\_113891 |
|  | NT\_167246 |
|  | NT\_167247 |
|  | NT\_167248 |
|  | NT\_167249 |
|  | NG\_029868 |
|  | AF001095 |
|  | AF065211 |
|  | AAD15888 |
|  | AF065212 |
|  | AAD15889 |
|  | AF208289 |
|  | AAG35728 |
|  | AF502127 |
|  | AJ012753 |
|  | CAA10169 |
|  | AJ238896 |
|  | CAB43094 |
|  | AL662830 |
|  | AL662884 |
|  | AL845464 |
|  | BX284686 |
|  | BX927239 |
|  | CH471081 |
|  | EAX03610 |
|  | EAX03611 |
|  | CR812478 |
|  | CR933878 |
|  | CS300822 |
|  | CAK32486 |
|  | D28769 |
|  | BAA05958 |
|  | KY500144 |
|  | AQY76648 |
|  | KY500145 |
|  | AQY76649 |
|  | KY500146 |
|  | AQY76650 |
|  | KY500147 |
|  | AQY76651 |
|  | KY500148 |
|  | AQY76652 |
|  | U89336 |
|  | AAB47491 |
|  | Y18060 |
|  | Y18061 |
|  | AB036432 |
|  | BAA89369 |
|  | AB061668 |
|  | BAC65465 |
|  | AB061669 |
|  | BAC65466 |
|  | AF536236 |
|  | AAQ10685 |
|  | AF536237 |
|  | AAQ10686 |
|  | AF537303 |
|  | AAQ10782 |
|  | AJ133822 |
|  | CAB43108 |
|  | AK298100 |
|  | BAG60385 |
|  | AK313178 |
|  | BAG35995 |
|  | AY755619 |
|  | AAX07272 |
|  | AY755620 |
|  | AAX07273 |
|  | AY755621 |
|  | AAX07274 |
|  | AY755622 |
|  | AAX07275 |
|  | AY755623 |
|  | AAX07276 |
|  | AY755624 |
|  | AAX07277 |
|  | AY755625 |
|  | AAX07278 |
|  | AY755626 |
|  | AAX07279 |
|  | AY755627 |
|  | AAX07280 |
|  | AY755628 |
|  | AAX07281 |
|  | BC020669 |
|  | AAH20669 |
|  | BM971899 |
|  | DQ104251 |
|  | AAZ32412 |
|  | DQ104252 |
|  | AAZ32413 |
|  | DQ104253 |
|  | AAZ32414 |
|  | DQ104254 |
|  | AAZ32415 |
|  | EU117141 |
|  | ABV03807 |
|  | EU428811 |
|  | ACE78167 |
|  | EU428812 |
|  | ACE78168 |
|  | EU428813 |
|  | ACE78169 |
|  | EU428814 |
|  | EU428815 |
|  | ACE78170 |
|  | EU428816 |
|  | EU826616 |
|  | ACF47652 |
|  | EU826617 |
|  | ACF47653 |
|  | EU826618 |
|  | ACF47654 |
|  | EU826619 |
|  | ACF47655 |
|  | EU826620 |
|  | ACF47656 |
|  | FJ545655 |
|  | ACL80027 |
|  | GU139370 |
|  | GU139371 |
|  | ADQ42256 |
|  | GU139372 |
|  | GU139373 |
|  | ADQ42257 |
|  | GU139374 |
|  | ADQ42258 |
|  | GU139375 |
|  | ADQ42259 |
|  | GU139376 |
|  | ADQ42260 |
|  | GU139377 |
|  | ADQ42261 |
|  | GU139378 |
|  | ADQ42262 |
|  | GU139379 |
|  | GU139380 |
|  | GU139381 |
|  | GU139382 |
|  | GU139383 |
|  | GU139384 |
|  | HQ148133 |
|  | ADV92535 |
|  | HQ148134 |
|  | ADV92536 |
|  | HQ148135 |
|  | ADV92537 |
|  | KC692917 |
|  | AHB30241 |
|  | M91211 |
|  | AAA03574 |
|  | Q15109 |
|  | NC\_000083 |
|  | XM\_006523501 |
|  | XP\_006523564 |
|  | NR\_073175 |
|  | NR\_073176 |
|  | NM\_001271424 |
|  | NP\_001258353 |
|  | NR\_073173 |
|  | NM\_007425 |
|  | NP\_031451 |
|  | NM\_001271422 |
|  | NP\_001258351 |
|  | NM\_001271423 |
|  | NP\_001258352 |
|  | NT\_039662 |
|  | NT\_187027 |
|  | NT\_187004 |
|  | AF030001 |
|  | AAB82007 |
|  | CH466666 |
|  | EDL26799 |
|  | EDL26800 |
|  | CT009767 |
|  | CU407310 |
|  | CU463834 |
|  | AB207883 |
|  | BAE72665 |
|  | AK004802 |
|  | BC061182 |
|  | AAH61182 |
|  | EU520325 |
|  | ACD35949 |
|  | EU570240 |
|  | ACE63492 |
|  | EU570241 |
|  | ACE63493 |
|  | EU570242 |
|  | ACE63494 |
|  | EU570243 |
|  | ACE63495 |
|  | EU570244 |
|  | ACE63496 |
|  | EU570245 |
|  | ACE63497 |
|  | EU570246 |
|  | ACE63498 |
|  | EU570247 |
|  | ACE63499 |
|  | EU906857 |
|  | ACK28143 |
|  | EU906858 |
|  | ACK28144 |
|  | EU906859 |
|  | ACK28145 |
|  | EU906860 |
|  | ACK28146 |
|  | EU906861 |
|  | ACK28147 |
|  | EU906862 |
|  | ACK28148 |
|  | EU906863 |
|  | ACK28149 |
|  | EU906864 |
|  | ACK28150 |
|  | GU164727 |
|  | ADX07277 |
|  | GU164728 |
|  | ADX07278 |
|  | GU164729 |
|  | ADX07279 |
|  | GU164730 |
|  | ADX07280 |
|  | GU164731 |
|  | ADX07281 |
|  | GU164732 |
|  | ADX07282 |
|  | GU164733 |
|  | GU164734 |
|  | GU164735 |
|  | GU164736 |
|  | GU164737 |
|  | GU164738 |
|  | GU164739 |
|  | KC692918 |
|  | AHB30242 |
|  | L33412 |
|  | AAA40040 |
|  | Q62151 |
|  | NC\_005119 |
|  | NM\_053336 |
|  | NP\_445788 |
|  | XM\_006256025 |
|  | XP\_006256087 |
|  | AC\_000088 |
|  | AABR07044388 |
|  | AAHX01099582 |
|  | BX883044 |
|  | CAE83960 |
|  | CH474121 |
|  | EDL83392 |
|  | EDL83393 |
|  | EDL83394 |
|  | BC084697 |
|  | AAH84697 |
|  | CR475487 |
|  | GU164715 |
|  | GU164716 |
|  | ADX07273 |
|  | GU164717 |
|  | ADX07274 |
|  | GU164718 |
|  | ADX07275 |
|  | GU164719 |
|  | ADX07276 |
|  | GU164720 |
|  | GU164721 |
|  | GU164722 |
|  | GU164723 |
|  | GU164724 |
|  | GU164725 |
|  | GU164726 |
|  | L33413 |
|  | AAA42027 |
|  | Q63495 |
|  | CAI41810 |
|  | CAI41811 |
|  | CAI41809 |
|  | CAM26225 |
|  | AC\_000039 |
|  | CAM26223 |
|  | CAM26224 |
|  | XP\_006536563 |
|  | ABM87820 |
|  | XP\_006525338 |
|  | DQ896821 |
|  | XP\_006537296 |
|  | ABBA01038108 |
|  | NC\_018917 |
|  | XM\_006537233 |
|  | CAQ06597 |
|  | CAQ06595 |
|  | CAQ06596 |
|  | XM\_006525275 |
|  | AABR06099863 |
|  | DQ893575 |
|  | AC\_000138 |
|  | CAM25716 |
|  | AAHY01132213 |
|  | XM\_006536500 |
|  | AMYH02013469 |
|  | ABM84501 |

---

|  |  |
| --- | --- |
| Swiss-Prot ID | RAGE\_HUMAN |
|  | RAGE\_MOUSE |

---

|  |  |
| --- | --- |
| Cell Localization | Cell membrane |
|  | Secreted |
|  | Membrane |
|  | Plasma membrane |

---

|  |  |
| --- | --- |
| Ensembl ID | ENSG00000204305 |
|  | ENSP00000364217.4 |
|  | ENST00000375076.9 |
|  | ENSP00000364210.4 |
|  | ENST00000375069.7 |
|  | ENSP00000387887.2 |
|  | ENST00000438221.6 |
|  | ENSP00000364195.2 |
|  | ENST00000375055.6 |
|  | ENSP00000364208.3 |
|  | ENST00000375067.7 |
|  | ENST00000484849.5 |
|  | ENSMUSG00000015452 |
|  | ENSMUSP00000134401.1 |
|  | ENSMUST00000174496.8 |
|  | ENSMUSP00000134579.1 |
|  | ENSMUST00000173992.7 |
|  | ENSMUSP00000015596.3 |
|  | ENSMUST00000015596.9 |
|  | ENSMUST00000174069.7 |
|  | ENSMUST00000173589.7 |
|  | ENSMUST00000172932.1 |
|  | ENSRNOG00000000439 |
|  | ENSRNOP00000000508.1 |
|  | ENSRNOT00000000508.4 |
|  | ENSRNOP00000072071.1 |
|  | ENSRNOT00000084841.1 |
|  | ENSMUST00000174069 |
|  | ENSRNOT00000084841 |
|  | ENSP00000364210 |
|  | ENST00000375055 |
|  | ENST00000375076.8 |
|  | ENSRNOP00000000508 |
|  | ENSRNOP00000072071 |
|  | ENSP00000364217 |
|  | ENSMUST00000174496 |
|  | ENST00000375067 |
|  | ENSMUSP00000134579 |
|  | ENST00000375069 |
|  | ENSMUSP00000015596 |
|  | ENSP00000364208 |
|  | ENSMUST00000173992 |
|  | ENST00000375076 |
|  | ENSMUSP00000134401 |
|  | ENSRNOT00000000508 |
|  | ENSP00000364195 |
|  | ENST00000438221 |
|  | ENSMUST00000015596 |
|  | ENSP00000387887 |
|  | ENSMUST00000173589 |
|  | ENSMUST00000172932 |
|  | ENST00000484849 |

---

|  |  |
| --- | --- |
| MGI ID | MGI:893592 |
|  | 893592 |

---

|  |  |
| --- | --- |
| RGD ID | 69258 |

---

|  |  |
| --- | --- |
| TCDB ID | 8.A.23.1.19 |

---

|  |  |
| --- | --- |
| Unigene ID | Hs.534342 |
|  | Mm.3383 |
|  | Rn.9829 |

---

|  |  |
| --- | --- |
| Homologene ID | 883 |

---

|  |  |
| --- | --- |
| Shape | Stick-vertex |

---

|  |  |
| --- | --- |
| IPI ID | IPI00014810 |
|  | IPI00431197 |
|  | IPI00220335 |

---
